# Multidrug Resistance in Neisseria gonorrhoeae: Identification of Functionally Important Residues in the MtrD Efflux Protein

**DOI:** 10.1128/mBio.02277-19

**Published:** 2019-11-19

**Authors:** Mohsen Chitsaz, Lauren Booth, Mitchell T. Blyth, Megan L. O’Mara, Melissa H. Brown

**Affiliations:** a College of Science and Engineering, Flinders University, Bedford Park, SA, Australia; b Research School of Chemistry, Australian National University, Canberra, ACT, Australia; Duke University School of Medicine

**Keywords:** *Neisseria gonorrhoeae*, multidrug resistance, efflux pumps, MtrCDE system, resistance-nodulation-division, molecular dynamics simulations, drug transport

## Abstract

A key mechanism that Neisseria gonorrhoeae uses to achieve multidrug resistance is the expulsion of structurally different antimicrobials by the MtrD multidrug efflux protein. MtrD resembles the homologous Escherichia coli AcrB efflux protein with several common structural features, including an open cleft containing putative access and deep binding pockets proposed to interact with substrates. A highly discriminating N. gonorrhoeae strain, with the MtrD and NorM multidrug efflux pumps inactivated, was constructed and used to confirm and extend the substrate profile of MtrD to include 14 new compounds. The structural basis of substrate interactions with MtrD was interrogated by a combination of long-timescale molecular dynamics simulations and docking studies together with site-directed mutagenesis of selected residues. Of the MtrD mutants generated, only one (S611A) retained a wild-type (WT) resistance profile, while others (F136A, F176A, I605A, F610A, F612C, and F623C) showed reduced resistance to different antimicrobial compounds. Docking studies of eight MtrD substrates confirmed that many of the mutated residues play important nonspecific roles in binding to these substrates. Long-timescale molecular dynamics simulations of MtrD with its substrate progesterone showed the spontaneous binding of the substrate to the access pocket of the binding cleft and its subsequent penetration into the deep binding pocket, allowing the permeation pathway for a substrate through this important resistance mechanism to be identified. These findings provide a detailed picture of the interaction of MtrD with substrates that can be used as a basis for rational antibiotic and inhibitor design.

## Abstract (precis)

With over 78 million new infections globally each year, gonorrhea remains a frustratingly common infection. Continuous development and spread of antimicrobial-resistant strains of Neisseria gonorrhoeae, the causative agent of gonorrhea, have posed a serious threat to public health. One of the mechanisms in N. gonorrhoeae involved in resistance to multiple drugs is performed by the MtrD multidrug resistance efflux pump. This study demonstrated that the MtrD pump has a broader substrate specificity than previously proposed and identified a cluster of residues important for drug binding and translocation. Additionally, a permeation pathway for the MtrD substrate progesterone actively moving through the protein was determined, revealing key interactions within the putative MtrD drug binding pockets. Identification of functionally important residues and substrate-protein interactions of the MtrD protein is crucial to develop future strategies for the treatment of multidrug-resistant gonorrhea.

## INTRODUCTION

The emergence of multidrug resistance (MDR) in bacteria is a global health problem that severely compromises effective treatment options, and efflux of antibiotics by membrane-bound transport proteins has been implicated as a key mechanism. In particular, in Gram-negative pathogens resistance-nodulation-division (RND) multidrug efflux systems represent the first line of defense for the cell. RND efflux systems constitute an inducible simultaneous MDR mechanism that acts against a broad spectrum of antibiotics and other antimicrobial agents. These tripartite protein complexes span the bacterial inner and outer membranes to provide a continuous drug efflux pathway.

The sexually transmitted pathogen Neisseria gonorrhoeae is unique among Gram-negative bacteria in that it contains a single RND efflux system to promote survival: the multiple transferable resistance (Mtr) system MtrCDE (1, 2). The MtrD efflux protein is embedded in the inner membrane and exports drugs from the periplasm and inner membrane to the MtrE outer membrane channel. MtrD contains the substrate binding sites and transduces the electrochemical energy required for drug export via a H^+^/substrate antiporter mechanism (3). MtrD and MtrE are anchored together by the periplasmic MtrC to enable substrate efflux from the bacterium.

MtrD shares 48.9% sequence identity with the homologous Escherichia coli RND exporter AcrB, which has provided the structural basis for our understanding of RND exporters to date. It is well established that homologous proteins share common structural features. However, the unique amino acid sequence of each protein allows recognition and efflux of a distinct set of compounds required for survival of that specific bacterial species in its particular environment. Similar to AcrB, MtrD effluxes a wide spectrum of compounds, including detergents, antibiotics, dyes, bile salts, fatty acids, biocides, steroidal hormones, and aliphatic and host-derived cationic antimicrobial peptides (4, 5). Given that gonococci commonly infect mucosal sites bathed in fluids containing a number of these compounds, MtrCDE contributes to bacterial virulence as well as to antimicrobial resistance, enhancing colonization and disease development (5, 6).

In 2014, the crystal structure of MtrD was resolved to 3.53 Å, revealing an architecture similar to those of the homologous AcrB and MexB structures (7). Each MtrD monomer shows two pseudosymmetric halves and contains two domains: a transmembrane domain (TM), which is organized into 12 transmembrane α-helices embedded in the cytoplasmic membrane, and a large periplasmic domain that can be subdivided into a porter and a docking domain. The porter domain is composed of four subdomains, i.e., PN1, PN2, PC1, and PC2. In MtrD, PN1 makes up the central pore and stabilizes the trimeric organization, while PC1 and PC2 create a substrate binding cleft along the pseudosymmetric axis between the two halves of the porter domain. The docking domain is formed of two subdomains, DN and DC, that likely interact with MtrE in the outer membrane (7, 8).

Structures of AcrB bound to various substrates have identified two multidrug-binding sites within the binding cleft of the porter domain: the proximal (access) and distal (deep) binding pockets, which are separated by a so-called “switch loop” (9, 10). It is postulated that substrates first enter the access pocket of the binding cleft and then permeate into the deep binding pocket. The AcrB deep binding pocket is rich in phenylalanine residues, and many residues that have been identified as crucial to the export process (F136, F178, F610, F615, F617, and F628) are highly conserved between MexB and MtrD, suggesting that they play important and possibly similar roles in MtrD.

To date, MtrD, has not been cocrystallized with a substrate. Thus, the residues that comprise the MtrD substrate binding site(s) and whether the substrate binding residues common to RND proteins also play a role in MtrD efflux have not been ascertained. In this study, we established a site-directed mutagenesis system specific for analyzing the MtrD export protein in its natural host and, using this system, we identified 14 new MtrD substrates and experimentally explored the role of F136, F176, I605, F610, S611, F612, and F623 in MtrD-mediated multidrug resistance. To characterize the structural basis of substrate interactions with MtrD, we used a combination of long-timescale molecular dynamics (MD) simulations and docking studies. Long-timescale MD simulations of progesterone showed the spontaneous binding of progesterone to the access pocket of the binding cleft and its subsequent permeation past the switch loop and into the deep binding pocket. Docking studies of progesterone, nonoxynol-9, azithromycin, rifampin, ethidium, crystal violet, cholic acid, and the RND pump inhibitor phenylalanine arginine beta-naphthylamide (PAβN) indicated that several key residues play important nonspecific roles in the efflux of these substrates.

## RESULTS

### A highly discriminating N. gonorrhoeae strain for *in situ* analysis of MtrD activity.

An expression and integration system was established in a N. gonorrhoeae host to analyze the function of wild-type (WT) MtrD and construct a set of isogenic mutants. This system has the advantage that it produces both cognate partner proteins of MtrD, i.e., MtrC and MtrE, allowing assembly of the active tripartite MtrCDE system in the neisserial membrane. This ensures that alterations in spectra and/or levels of resistance would be solely due to manipulation of MtrD. The well-characterized antibiotic-sensitive strain N. gonorrhoeae FA19 was selected as the background strain (11). As an initial step, three isogenic FA19 derivatives (FA19 Δ*mtrD*, FA19 Δ*norM*, and FA19 Δ*mtrD* Δ*norM* mutants) were created using overlap extension PCR (12). The NorM efflux pump of N. gonorrhoeae is known to expel a number of cationic toxic compounds, such as ciprofloxacin, ethidium, and acriflavine; the latter two compounds are substrates common with MtrD (13). Thus, to remove any contribution that the unrelated NorM multidrug efflux pump would have provided in the assessment of MtrD function, the MtrD derivatives were constructed in a strain in which *norM* was also deleted from the chromosome. To achieve a high level of discrimination for antimicrobial susceptibility assessments of MtrD, the isogenic FA19 derivate KH15 was also employed. This strain of N. gonorrhoeae has a single-base-pair deletion in the *mtrR* promoter that results in upregulation of the *mtrCDE* system and consequential increased levels of resistance (5). Hence, two KH15 isogenic derivatives (KH15 Δ*mtrD* and KH15 Δ*mtrD* Δ*norM* mutant strains) were also constructed using the same overlap extension PCR methodology. Sequencing of the complete *mtrRCDE* region of the KH15 Δ*mtrD* and KH15 Δ*mtrD* Δ*norM* strains confirmed deletion of the *mtrD* gene and ensured the integrity of the *mtrR*/*C* promoter region as well as that of the *mtrC* and the *mtrE* genes. Resistance profiles of the resulting strain set were determined for 33 compounds, which included 2 known RND pump inhibitors (Tables 1, 2, and 3).

**TABLE 1:** Antimicrobial resistance profiles of Neisseria gonorrhoeae strains for 11 compounds^*a*^

Strain or mutant	MIC (μg/ml)										
	Detergent					Antibiotic					
	N-9	TX-100	SDS	TW-80	CHAPS	RIF	NOV	ERY	AZM	CRO	OXA
N. gonorrhoeae FA19	64	128	16	64	2,048	0.03	0.25	0.25	0.125	0.0005	0.50
N. gonorrhoeae FA19 Δ*mtrD*	16	32	16	64	1,024	0.008	0.03	0.06	0.03	0.0005	0.06
N. gonorrhoeae FA19 Δ*norM*	64	128	16	64	2,048	0.03	0.25	0.25	0.125	0.0005	0.50
N. gonorrhoeae FA19 Δ*mtrD* Δ*norM*	16	32	16	64	1,024	0.008	0.03	0.06	0.03	0.0005	0.06
N. gonorrhoeae KH15	≥4,096	≥4,096	32	128	>2,048	0.125	1	2	0.50	0.001	4
N. gonorrhoeae KH15 Δ*mtrD*	16	32	16	64	1,024	0.008	0.03	0.06	0.03	0.0005	0.06
N. gonorrhoeae KH15 Δ*mtrD* Δ*norM*	16	32	16	64	1,024	0.008	0.03	0.06	0.03	0.0005	0.06
N. gonorrhoeae KH15 Δ*mtrD* Δ*norM*(*mtrD*)	≥4,096	≥4,096	32	128	>2,048	0.125	1	ND	ND	0.001	4

a All MIC data are representative of three or more independent experiments. Abbreviations: AZM, azithromycin; CHAPS, 3-[(3-cholamidopropyl)dimethylammonio]-1-propanesulfonate; CRO, ceftriaxone; ERY, erythromycin; N-9, nonoxynol-9; ND, not determined; NOV, novobiocin; OXA, oxacillin; RIF, rifampin; TW-80, Tween 80; TX-100, Triton X-100.

**TABLE 2:** Antimicrobial resistance profiles of Neisseria gonorrhoeae strains for an additional 11 compounds^*a*^

Strain or mutant	MIC (μg/ml)										
	Antibiotic						CAMP		Dye		
	CHL	TET	PEN	TGC	CIP	GEN	CST	PMB	ET	CV	ACR
N. gonorrhoeae FA19	0.75	0.25	0.024	0.06	0.003	4	200	100	2	1	0.5
N. gonorrhoeae FA19 Δ*mtrD*	0.5	0.25	0.016	0.03	0.003	4	100	50	0.5	0.125	0.25
N. gonorrhoeae FA19 Δ*norM*	0.5	0.25	0.024	0.06	0.003	4	200	100	0.25	1	0.25
N. gonorrhoeae FA19 Δ*mtrD* Δ*norM*	0.5	0.25	0.016	0.03	0.0025	4	100	50	0.06	0.125	0.06
N. gonorrhoeae KH15	0.75	0.25	0.060	0.125	0.003	4	400	100	4	2	0.5
N. gonorrhoeae KH15 Δ*mtrD*	0.5	0.25	0.016	0.03	0.003	4	100	50	0.5	0.125	0.25
N. gonorrhoeae KH15 Δ*mtrD* Δ*norM*	0.5	0.25	0.016	0.03	0.0025	4	100	50	0.06	0.125	0.06
N. gonorrhoeae KH15 Δ*mtrD* Δ*norM*(*mtrD*)	>0.75	0.25	0.060	0.125	0.003	4	400	100	0.25	2	0.125

a All MIC data are representative of three or more independent experiments. Abbreviations: ACR, acriflavine; CHL, chloramphenicol; CIP, ciprofloxacin; CST, colistin; CV, crystal violet; ET, ethidium; GEN, gentamicin; PEN, benzylpenicillin; PMB, polymyxin B; TET, tetracycline; TGC, tigecycline.

**TABLE 3:** Antimicrobial resistance profiles of Neisseria gonorrhoeae strains for 11 more compounds^*a*^

Strain or mutant	MIC (μg/ml)										
	Bile acid	Fatty acid		Biocide					Hormone	Inhibitor	
	CHO	CA	PA	BC	CH	TR	DQ	PT	PRO	NMP	PAβN
N. gonorrhoeae FA19	200	12.5	12.5	2	0.25	0.125	4	4	40	ND	ND
N. gonorrhoeae FA19 Δ*mtrD*	100	12.5	6.25	1	0.125	0.06	2	2	20	ND	ND
N. gonorrhoeae FA19 Δ*norM*	200	12.5	12.5	2	0.25	0.125	1	1	40	ND	ND
N. gonorrhoeae FA19 Δ*mtrD* Δ*norM*	100	12.5	6.25	1	0.125	0.06	1	1	20	ND	ND
N. gonorrhoeae KH15	400	25	100	4	0.5	0.25	16	8	80	256	512
N. gonorrhoeae KH15 Δ*mtrD*	100	12.5	6.25	1	0.125	0.06	1	2	20	64	128
N. gonorrhoeae KH15 Δ*mtrD* Δ*norM*	100	12.5	6.25	1	0.125	0.06	1	1	20	64	128
N. gonorrhoeae KH15 Δ*mtrD* Δ*norM*(*mtrD*)	400	25	ND	4	0.5	0.25	1	1	80	256	512

a All MIC data are representative of three or more independent experiments. Abbreviations: BC, benzalkonium; CA, capric acid; CH, chlorhexidine; CHO, cholic acid; DQ, dequalinium; NMP, 1-(1-naphtylmethyl)-piperazine; PA, palmitic acid; PAβN, phenylalanine arginine beta-naphthylamide; PRO, progesterone; PT, pentamidine; TR, triclosan.

Deletion of *mtrD* and *norM* from FA19 resulted in a (1.5-fold to 32-fold) reduction of the MICs for 25 of 31 of the tested compounds (Tables 1, 2, and 3), and 29 of 31 compounds showed reduced (1.5-fold to >256-fold) MIC levels in the KH15 strain background. SDS, Tween 80, capric acid, and ceftriaxone showed a decrease in resistance only in the KH15 background, probably due the higher level of *mtrCDE* expression in this strain than in strain FA19 (Tables 1 and 3). In fact, a 4-fold or greater reduction in MICs was observed for 20 of 31 tested compounds for the KH15 derivatives, while this level of reduction of drug MICs in the FA19 background was observed for only 9 compounds. These data suggested that the KH15 Δ*mtrD* Δ*norM* strain can function as a good discriminative background strain for conducting analyses of *mtrD* mutants.

No change in resistance profiles was observed for the antibiotics tetracycline and gentamicin in strains with either *mtrD* or *norM* or both inactivated (Table 2). Although tetracycline has previously been identified as an MtrD substrate, this can be observed only in strains with concomitant mutations in multiple genes, including *mtrR*; these were not present in our background strain (14–16).

In addition to antimicrobial compounds, N. gonorrhoeae KH15 and derivatives were tested against two known RND pump inhibitors, PAβN and 1-(1-naphtylmethyl)-piperazine (NMP), for their resistance capacities. These assays showed decreased resistance of KH15 Δ*mtrD* and KH15 Δ*mtrD* Δ*norM* cells to PAβN and NMP, with 8-fold and 4-fold reductions in MICs, respectively (Table 3). Deletion of *norM* from the KH15Δ*mtrD* strain did not result in a further decrease of MIC for either compound. Thus, inactivation of *mtrD* in KH15 produced a decrease in resistance to 31 compounds, confirming the broad spectrum of antimicrobials that can be handled by this protein (Tables 1, 2, and 3).

### Function can be restored in the N. gonorrhoeae KH15 Δ*mtrD* Δ*norM* derivative by reinsertion of *mtrD*.

A procedure was established for introduction of *mtrD* derivatives, all containing a sequence encoding six C-terminal histidine residues (*mtrD*_His6_) into the chromosome of the KH15 Δ*mtrD* Δ*norM* strain via the pGCC4 (*Neisseria* insertion complementation system [NICS]) shuttle vector. This system allows integration of an *mtrD* determinant into the N. gonorrhoeae chromosome between *aspC* and *lctP* and provides cells with a recombinant *mtrD* allele with its expression under *lac* promoter regulation (17). A pGCC4-*mtrD*_(His6)_ clone was constructed and used for recombination of the WT *mtrD* allele into the N. gonorrhoeae KH15 Δ*mtrD* Δ*norM* chromosome, producing the KH15 Δ*mtrD* Δ*norM*(*mtrD*_His6_) strain. At the same time, the pGCC4 empty vector was also recombined to create an isogenic KH15 Δ*mtrD* Δ*norM*(NICS) strain that contained all the NICS elements, including the erythromycin resistance determinant present on the pGCC4 vector used for selection, and that could be utilized as a negative/background control. Western blotting confirmed expression and localization of the recombinant WT MtrD_His6_ protein within isolated membranes of N. gonorrhoeae KH15 Δ*mtrD* Δ*norM*(*mtrD*_His6_) cells (see Fig. S1 in the supplemental material).

10.1128/mBio.02277-19.2 FIG S2 Protein sequence alignment of MtrD (GenBank accession no. AAC45560.1), AcrB (GenBank accession no. P31224), and MexB (GenBank accession no. P52002). Protein sequence alignment was done using the T-Coffee program and visualized by BoxShade with residue boxes colored based on percent identity. MtrD residues targeted by site-directed mutagenesis are shown within green rectangles with the residue number at the top in red. Download FIG S2, DOCX file, 0.2 MB. Copyright © 2019 Chitsaz et al. 2019 Chitsaz et al. This content is distributed under the terms of the Creative Commons Attribution 4.0 International license.

Complementation of the KH15 Δ*mtrD* Δ*norM* strain with *mtrD*_His6_ restored resistance to WT levels fully for 24 compounds and partially for 4 compounds (Tables 1, 2, and 3). Partial complementation was seen for ethidium, acriflavine, dequalinium, and pentamidine, as the MICs were below those for the parental strain (Tables 2 and 3). This was expected, as these four compounds are substrates of both the MtrD and NorM multidrug efflux pumps. These data confirmed that the MtrCDE system is functional in the KH15 Δ*mtrD* Δ*norM*(*mtrD*_His6_) strain, offering a reliable system for analyzing incorporated site-directed MtrD mutants. Additionally, these data showed that the histidine-tagged MtrD protein in the KH15 Δ*mtrD* Δ*norM*(*mtrD*_His6_) strain provides resistance to a large number of MtrD substrates, allowing selection of a range of structurally different compounds for functional analysis of MtrD mutants. From these analyses, 11 compounds were chosen as representative compounds for subsequent MIC analysis of MtrD activity due to their high discrimination in MIC analysis and provide examples of a range of antimicrobials from different chemical classes. While both erythromycin and azithromycin demonstrated excellent MIC differentiation between the *mtrD* deletion strain and the corresponding parental strain, the use of the erythromycin resistance cassette present in pGCC4 as a selection marker precluded testing constructed MtrD mutant derivatives against these clinically important antibiotics.

### Spontaneous uptake of progesterone identified from MD simulations.

**FIG 1:**
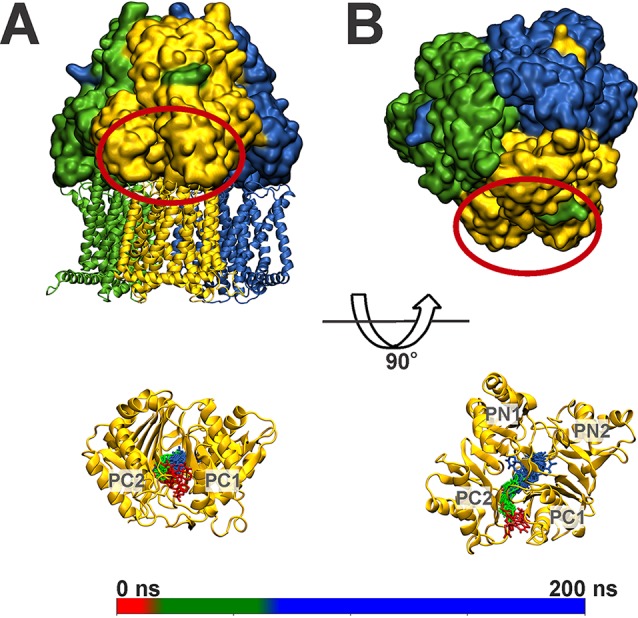
Progesterone binding in monomer B of the MtrD trimer shown as a (A) side view and (B) top view. Monomers A, B, and C of MtrD are colored green, gold, and blue, respectively. The periplasmic region of monomer B is circled in red. The lower panels show MtrD monomer B with the position of progesterone throughout the 200-ns simulation. Snapshots of progesterone taken every 10 ns are shown in licorice representation and colored according to simulation time. The scale bar gives the correlation between simulation time and progesterone color. The starting position of the progesterone molecule at 0 ns is shown in red, and the final position of progesterone at 200 ns, straddling the switch loop, is in blue.

**FIG 2:**
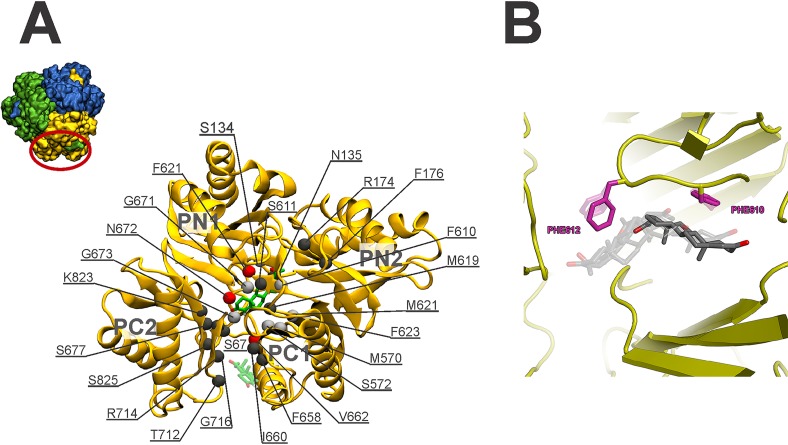
(A) Porter domain of MtrD viewed from the top, illustrating the residues implicated in binding of progesterone. Progesterone is drawn in licorice representation at 0 ns (washed out) and 200 ns. The Cα atoms of 25 residues most frequently within 4 Å of progesterone are drawn in van der Waals representation and colored as follows: red, >75% frequency; silver, >50% frequency; dark gray, >25% frequency. The inset shows a top view of the MtrD trimer with the periplasmic region of monomer B in gold. (B) The lowest-energy docked poses for progesterone in the access and deep pockets. F610 (magenta) plays the largest role in binding and transport of this substrate in the access pocket. F612 (magenta) plays the largest role in binding and transport of this substrate in the deep pocket.

To investigate the interaction of MtrD with substrates, spontaneous binding simulations were performed with the substrate progesterone. In these simulations, 30 molecules of progesterone were randomly placed in the aqueous solution surrounding the MtrD porter and docking domains. In two of the three simulations (representing 700 ns of simulation time), progesterone randomly adsorbed to the protein via nonspecific interactions. In the remaining 200-ns replicate, a single molecule of progesterone entered the access pocket of the binding cleft, straddled the switch loop, and in the first 100 ns of the simulation interacted with the deep binding pocket, where it remained for the duration of the analysis. The time-dependent motion of progesterone within the binding cleft throughout the simulation is shown in Fig. 1. In the first 2 ns of the simulation, progesterone associated with the outer lip of the binding cleft in an upright orientation. Progesterone then rotated 90° to interact with the β-sheet residues from PC2 that line the binding cleft (Fig. 1, bottom panel, red) before moving into the MtrD access binding pocket, where it resided for the first 40 ns of the simulation (Fig. 1, bottom panel, green). After 40 ns of the simulation, progesterone moved further into the binding cleft, straddling the switch loop, before interacting with the deep binding pocket, where it remained for the rest of the 200-ns trajectory. Throughout the last 100 ns of the simulation, progesterone moved freely and changed orientation in the deep binding pocket, residing ∼10 Å from the entrance of the binding cleft. From these simulations, 25 binding cleft residues were identified as being within 4 Å of progesterone throughout the simulation (Fig. 2). These residues include four phenylalanines (F174, F610, F612, and F623) that are conserved in both AcrB and MexB (Table 4; see also Fig. S2).

**TABLE 4:** Residues belonging to binding pockets of MtrD and AcrB

Region	AcrB residues	MtrD residues from alignment^*a*^
Access pocket	79, 91, 134, 135, 292, 573, 575, 577, 617, 624, 662, 664, 666, 667, 668, 674, 676, 681, 717, 719, 826	79, 91, **134**, **135**, 290, **570**, **572**, 574, **612**, **619**, **658**, **660**, **662**, 663, 664, 670, **672**, 678, **714**, **716**, **823**

Deep binding pocket	46, 89, 128, 130, 134, 136, 176, 177, 178, 180, 273, 274, 276, 277, 290, 327, 573, 610, 612, 615, 617, 620, 628	46, 89, 128, 130, **134**, 136, **174**, 175, **176**, 178, 271, 272, 274, 275, 288, 325, **570**, 605, 607, **610**, **612**, 615, **623**

a MtrD residues implicated in binding progesterone in MD simulations are shown in bold; residues contributing to both the access and deep binding pockets are underlined.

10.1128/mBio.02277-19.1 FIG S1 An example picture of a Western blot (WB) showing expression levels of the recombinant wild-type MtrD (WT) and seven MtrD mutants (labeled on the top of each lane) in isolated membranes of the KH15 *ΔmtrD* Δ*norM* strain expressing these mutants. The negative (–ve) control is the same strain complemented with the NotI-cut NICS part of the pGCC4 vector lacking the *mtrD* gene. Download FIG S1, DOCX file, 0.2 MB. Copyright © 2019 Chitsaz et al. 2019 Chitsaz et al. This content is distributed under the terms of the Creative Commons Attribution 4.0 International license.

### Docking of substrates to MtrD.

The use of MD simulation studies is a computationally expensive approach for analysis of the interaction of MtrD with a range of substrates. Thus, to determine whether a simplified docking approach could give insights into key MtrD residues in contact with substrates, flexible docking studies of progesterone were compared with MD simulations. Progesterone docked into the deep binding pocket of MtrD in several orientations, as shown in Fig. 2B, primarily interacting with F623, F612, F610, F176, and F136. A number of these residues were also highlighted in MD simulations; for example, progesterone interacted with F612 for >90% of the simulation time, while F623 and F610 interacted with progesterone for >50% of the simulation time, and the interaction with F176 persisted for 25% of the simulation time.

Overall, docking studies and MD simulations of progesterone binding were in good agreement. Thus, we extended the procedure to dock a further six substrates and one inhibitor into the MtrD access and deep binding pockets. These compounds were the substrates nonoxynol-9, azithromycin, rifampin, ethidium, crystal violet, and cholic acid and the inhibitor PAβN. As stated previously, we were unable to test resistance to azithromycin for the MtrD mutants in our *in vivo* system. Thus, the ability to analyze the interaction of MtrD with azithromycin through *in silco* docking studies provides an example of the usefulness and versatility of this system. The lowest-energy docked poses revealed a set of common residues that appear to play a role in the binding and/or transport of these compounds (Fig. S3 to S6). Critically, a number of residues (F136, F176, F610, F612, and F623) were found to be implicated in substrate binding and are conserved in both the AcrB and MexB efflux proteins (Fig. S3 to S6). Site-directed mutagenesis was used to confirm their importance in MtrD-mediated resistance.

10.1128/mBio.02277-19.3 FIG S3 (A) The lowest-energy docked poses for crystal violet. Three residues, F612, F136, and F610 (magenta sticks), form key interactions with crystal violet in all of the lowest-energy docked conformations. (B) The lowest-energy docked poses for ethidium. Residues F610, F612, and F136 (magenta sticks) interact with the lowest-energy conformations of ethidium docked to either the access pocket or the deep binding pocket of MtrD. Download FIG S3, DOCX file, 2.1 MB. Copyright © 2019 Chitsaz et al. 2019 Chitsaz et al. This content is distributed under the terms of the Creative Commons Attribution 4.0 International license.

### Site-directed mutagenesis identified six residues involved in MtrD-mediated resistance.

Detailed knowledge of the interactions between substrates and amino acids that comprise drug binding sites within the porter domain of AcrB has been obtained by a variety of methods (18–20). Such studies identified, among others, a cluster of six phenylalanine residues (F136, F178, F610, F615, F617, and F628) that contribute to formation of the deep drug binding pocket and that are involved in substrate binding and recognition (9, 10). In MtrD, five of these phenylalanine residues are conserved (F136, F176, F610, F612, and F623) at similar locations, whereas F610 is an isoleucine in the corresponding position in MtrD (I605) (Fig. S2). Also included in our set of targets was S611, corresponding to G616 in AcrB (Fig. S2), which has been identified as playing a role in resistance (18, 21, 22). Thus, we were interested to learn whether these residues perform similar roles in the related RND transporter MtrD.

To facilitate later studies on the locations of substituted residues and on their inhibition (23), we initially generated a cysteineless variant of the *mtrD*_His6_ clone in pGCC4 (pGCC4-CL*mtrD*_His6_). This produced a MtrD C491A variant in which the sole cysteine residue at position 491 was replaced with alanine. When integrated into the KH15 Δ*mtrD* Δ*norM* background, the KH15 Δ*mtrD* Δ*norM*(CL*mtrD*_His6_) strain had WT levels of resistance to all tested compounds (Table 5), and Western blot expression levels of the CL-MtrD_His6_ protein in neisserial membranes were comparable to the expression level measured for WT MtrD_His6_ (Fig. S1). As a result, the MtrD mutants described below were constructed in this pGCC4-CL*mtrD*_His6_ derivative. Seven MtrD mutants (F136A, F176A, I605A, F610A, S611A, F612C, and F623C) were generated and individually recombined into the N. gonorrhoeae KH15 Δ*mtrD* Δ*norM* chromosome. Western blotting of isolated membranes from these bacteria confirmed expression of the MtrD mutant proteins at levels comparable to that determined for the CL-MtrD_His6_ parent (Fig. S1).

**TABLE 5:** Neisseria gonorrhoeae KH15 *ΔmtrD ΔnorM* strain expressing MtrD derivatives

Mutation	MIC (μg/ml)^*a*^										
	Detergent		Antibiotic			CAMP	Dye		Bile acid	Hormone	Biocide
	N-9	TX-100	RIF	NOV	OXA	PMB	ET	CV	CHO	PRO	DQ
MtrD_His6_^*b*^	≥4,096	≥4,096	0.125	1	4	100	0.25	2	400	80	1
CL-MtrD_His6_	≥4,096	≥4,096	0.125	1	4	100	0.25	2	400	80	1
F136A	≥4,096	≥4,096	0.03	0.25	2	100	0.25	0.5	200	80	1
F176A	32	128	0.03	0.125	0.5	50	0.06	0.25	200	20	0.5
I605A	32	128	0.03	0.5	1	100	0.125	1	400	80	0.5
F610A	≥4,096	≥4,096	0.125	0.5	4	100	0.25	2	400	80	1
S611A	≥4,096	≥4,096	0.125	1	4	100	0.25	2	400	80	1
F612C	32	128	0.016	0.125	0.5	50	0.06	0.25	200	20	0.5
F623C	16	64	0.016	0.125	0.25	50	0.06	0.125	100	20	0.5
Negative control^*c*^	16	32	0.008	0.03	0.06	50	0.06	0.125	100	20	0.5

a All MIC data are representative of three or more independent experiments. Abbreviations: CAMP, cationic antimicrobial peptide; CHO, cholic acid; CV, crystal violet; DQ, dequalinium; ET, ethidium; N-9, nonoxynol-9; NOV, novobiocin; OXA, oxacillin; PMB, polymyxin B; PRO, progesterone; RIF, rifampin; TX-100, Triton X-100.

b Recombinant WT MtrD_His6_.

c KH15 *ΔmtrD ΔnorM* strain transformed with pGCC4 empty vector expressing no MtrD [KH15 Δ*mtrD* Δ*norM*(NICS) strain].

Three MtrD mutants (F176A, F612C, and F623C) exhibited reduced resistance to all 11 compounds, indicating a significant and wide-ranging impact on resistance (Table 5). No resistance to ethidium, polymyxin B, progesterone, and dequalinium was observed for any of the three mutants, and F623 showed only background levels of resistance to nonoxynol-9. They did retain a small degree of resistance to the other six compounds, namely, Triton X-100, rifampin, novobiocin, crystal violet, oxacillin, and cholic acid (Table 5). It is worth noting that the retention of this small degree of resistance is evidence that the complete loss of resistance that was seen for the group of four substrates was likely not due to complete misfolding of the mutant protein. These data suggest that the F176, F612, and F623 residues play important and widespread roles in MtrD-mediated multidrug resistance.

Replacement of residues in MtrD at positions 136, 605, and 610 differentially affected resistance to distinct compounds (Table 5). The MtrD F136A mutant showed reduced resistance to five compounds, while no changes in MICs were observed for six compounds (Table 5). Substitution of I605 with alanine reduced resistance to 8 of 11 tested compounds, with the WT resistance profile retained for only 3 compounds (Table 5). The effect of substitution of F610 was more limited, as MtrD F610A showed a reduction in resistance only to novobiocin, which was demonstrated by a consistent 2-fold reduction in MICs for this compound (Table 5). The MtrD S611A mutant was the only variant that retained a complete WT resistance profile with no change in MICs for any tested compound (Table 5).

## DISCUSSION

Antibiotic resistance in N. gonorrhoeae is an effective survival strategy, as this bacterium has been successful in developing resistance to almost all antibiotics previously or currently used for treatment of gonorrhea. With the emergence and spread of gonococci strains that have shown resistance to the last options of empirical therapy, azithromycin and ceftriaxone, the problem has become more alarming. The limited discovery of new antibiotics during past 3 decades, particularly of those against Gram-negative bacteria, has intensified this concern. One of the mechanisms of drug resistance utilized by N. gonorrhoeae involves efflux pumps that enable the cells to keep interior levels of drugs below toxic levels. Recent reports have highlighted the importance of this mechanism of resistance by showing that N. gonorrhoeae can acquire mosaic drug efflux gene sequences from commensal *Neisseria* that can lead to low-level azithromycin resistance expressed by N. gonorrhoeae clinical isolates (24). Interestingly, the acquired mosaic-like sequence within *mtrD* in these isolates was found to increase MtrD activity, resulting in clinical resistance to azithromycin in the background of elevated expression of the MtrCDE efflux proteins.

To analyze in detail the MtrD pump component of the MtrCDE efflux system, a site-directed mutagenesis system specific for manipulation and analysis of this protein in its natural host, N. gonorrhoeae, was established. Addition of a histidine tag on the C terminus of MtrD for monitoring expression levels and increasing the versatility of the system did not affect resistance (Tables 1, 2, and 3), making the MtrD_His6_ protein advantageous for studying the effect of mutations on resistance to many important antibiotics, biocides, and human-derived antimicrobial peptides. Additionally, the cysteineless derivative of MtrD_His6_ (CL-MtrD_His6_) retained full activity compared to the corresponding parental MtrD_His6_ protein, providing a reliable site-directed cysteine-scanning mutagenesis system for further analysis of MtrD (Table 5).

This study revealed that the MtrD efflux pump possesses an even wider substrate specificity than had previously been proposed (5, 15, 25) with the addition of 14 new substrates, including detergents (SDS, CHAPS {3-[(3-cholamidopropyl)dimethylammonio]-1-propanesulfonate}, and Tween 80); antibiotics (tigecycline, novobiocin, ciprofloxacin, and pentamidine); biocides (dequalinium, benzalkonium, chlorhexidine, and triclosan); and a cationic polypeptide (colistin) as well as two efflux pump inhibitors PAβN and NMP (Tables 1, 2, and 3).

One of the interesting features of RND transporters is that they recognize and export a remarkably broad range of substrates from different chemical classes. This feature has been mainly attributed in AcrB to the existence of multiple pockets with each pocket containing multiple overlapping drug-binding sites within the porter domain as well as several entrances with specificity for different substrates (18, 26, 27), similarly to the substrate recognition mechanism first described for the multidrug binding protein QacR (28, 29). Additionally, the presence of multiple aromatic residues such as phenylalanine in a multisite substrate binding pocket that can act in a drug-specific manner has been reported previously for both QacR and AcrB (28). Similar features are apparent in the MtrD polyspecific RND efflux transporter. MD simulations of progesterone binding to MtrD identified 25 residues within the binding cleft that are implicated in progesterone binding (Table 4; see also Fig. 2A). This was in good agreement with docking results from analysis of progesterone, which identified a similar set of residues in contact with progesterone, including F176, F610, F612, and F623. Docking studies of a further six compounds also indicated that these five phenylalanine residues form key interactions with substrates in the access and deep binding pockets of MtrD. Mutational analyses of these five phenylalanine residues showed reduced resistance to various MtrD substrates, confirming the integral role that these aromatic residues play in MtrD drug binding and translocation.

Progesterone binding MD simulations of MtrD, docking analyses of eight compounds, and MIC analysis of F176A and F623C mutants showed that these residues, located in the region of MtrD corresponding to the deep binding pocket, have important functional roles in drug binding and resistance. This is similar to what has been ascribed to the homologous residues in AcrB, F178 and F628, which are involved in binding to drugs and RND efflux pump inhibitors (9, 26, 30–37).

F612 is a conserved residue located in the region corresponding to the AcrB switch loop (F617 in ArcB) that projects into the binding cleft cavity between the access and deep binding pocket. During the transition from the access conformation to the binding conformation of AcrB, movement of the switch loop allows high-molecular-mass (*M_r_* > 600 dalton) substrates to move from the AcrB access pocket to the deep binding pocket (10, 38). Spontaneous binding of progesterone from MD simulations showed that Cα of F612 was within 4 Å of progesterone for >75% of the 200-ns simulation time (Table 4; see also Fig. 2A), while docking analyses confirmed that F612 is a contributor to binding affinity for all eight docked compounds (Fig. 2B; see also Fig. S3 to S6 in the supplemental material). Analysis of cells expressing the F612C MtrD mutant showed a significant reduction in resistance to all 11 tested compounds, confirming the importance of F612 in resistance afforded by MtrD (Table 5).

Like the homologous counterparts in AcrB (F136, F610, and F615), F136, I605, and F610 form part of the deep binding pocket and are involved in nonspecific drug binding of MtrD (Fig. S2) (9, 32, 37, 39). Our progesterone binding MD simulations showed that Cα of MtrD F610 was within 4 Å of progesterone for >50% of the simulation time (Fig. 2A). Docking analyses identified F136 and F610 as contributors to the binding affinity for various compounds (Fig. 2B; see also Fig. S3 to S6), and the data were confirmed by MIC analysis of the corresponding MtrD mutant derivatives, even though the MIC for progesterone was unaffected in the F610A mutant. MIC data also suggest that I605, located in the distal region of the deep binding pocket, has a functionally important role in MtrD. However, analysis of the starting MtrD crystal structure and MD trajectories showed that, unlike the corresponding residue in AcrB (F610), I605 did not form part of the solvent-accessible cavity surface of the deep binding pocket in the MtrD crystal structure and thus did not interact with progesterone in either MD simulations or docking-based studies. The lack of correlation between the MIC data and the cocrystallography or MD simulation data is not uncommon in studies of these dynamic transporters, which undergo large-scale conformational changes during their transport cycle.

The use of MD simulations of progesterone binding in this study was found to be a successful approach for characterization of MtrD binding to a substrate. The predictive power of this method comes from a circumstantial finding of this study, which identified K823, located in the access pocket, as interacting with progesterone. In a recent study of a N. gonorrhoeae clinical isolate possessing a mosaic-like *mtr* efflux pump locus with reduced susceptibility to antimicrobials, mutation of K823 to glutamate (K823E) resulted in a gain-of-function impact on MtrD activity (40) suggesting an important functional role for this residue in MtrD drug translocation and confirming our assignment of this residue in substrate binding.

Characterization of antimicrobial efflux mechanisms in bacteria has helped understanding of the resources through which bacteria, including N. gonorrhoeae, are able to develop resistance against toxic compounds present in their environment, including those found naturally and antibiotics used for treatment of infections. In Gram-negative bacteria in particular, characterization of RND efflux pumps, including the gonoccocal MtrCDE system, has helped improve understanding of the main barrier for accumulation of antibiotics within cells at toxic levels. The detailed molecular, biochemical, structural and computational studies of RND pumps, including the N. gonorrhoeae MtrD protein, have provided new insights regarding determinations of operation/efflux mechanisms, substrate pathways, amino acid residues required for drug recognition and binding, and residues that are involved in other mechanisms of efflux operation such as energy coupling or interaction with partner proteins. These studies have also provided new insights regarding how some molecules interact with and inhibit RND pumps. New drugs that bypass efflux mechanisms are desperately needed for treatment of infections with highly resistant bacterial strains, especially of infections with Gram-negative bacteria. Identification of functionally important residues in MtrD represents a start for efforts aimed at gaining a better understanding of the transport mechanism(s) of the resistance-nodulation division family of multidrug transporters and at rationally based designing of antimicrobial drugs or efflux pump inhibitors.

## MATERIALS AND METHODS

### Bacterial strains, plasmids, and growth conditions.

The bacterial strains and plasmids used in this study are listed in Table 6. Gonococci were grown on GC medium (Difco Laboratories, Detroit, MI) as previously described (41). The pGCC4 vector was used as the backbone in which *mtrD* mutants were constructed and then mobilized into N. gonorrhoeae (see below). Erythromycin was added into GC plates for selecting N. gonorrhoeae transformed with pGCC4 (0.5 μg/ml) or for replica plating for selection of *mtrD* deletions (0.06 μg/ml). Isopropyl-β-d-thiogalactopyranoside (IPTG) was used at a final concentration of 0.5 mM to induce *mtrD* expression in N. gonorrhoeae strains containing integrated *mtrD* constructs.

**TABLE 6:** Bacterial strains and plasmids used in this study

Strain or plasmid	Relevant genotypes or description^*a*^	Source or reference
Strains		
E. coli DH5α	*fhuA2* Δ(*argF*-*lac*Z)*U169 phoA glnV44* Φ80 Δ(*lacZ*)M15 *gyrA96 recA1 relA1 endA1 thi-1 hsdR17*	42
N. gonorrhoeae FA19	Antimicrobial sensitive	15
N. gonorrhoeae KH15	As FA19 but *mtrR*-171	5
N. gonorrhoeae FA19 Δ*mtrD*	As FA19 but Δ*mtrD*	This study
N. gonorrhoeae FA19 Δ*norM*	As FA19 but Δ*norM*	This study
N. gonorrhoeae FA19 Δ*mtrD* Δ*norM*	AS FA19 but Δ*mtrD* and Δ*norM*	This study
N. gonorrhoeae KH15 Δ*mtrD*	AS KH15 but Δ*mtrD*	This study
N. gonorrhoeae KH15 Δ*mtrD* Δ*norM*	AS KH15 but Δ*mtrD* and Δ*norM*	This study
N. gonorrhoeae KH15 Δ*mtrD* Δ*norM*(*mtrD*)	As KH15 Δ*mtrD* Δ*norM* but complemented with WT *mtrD*_6his_	This study

Plasmids		
pGCC4	*Neisseria* insertion complementation system (NICS) vector; *lacI*, P_lac_, Kan^r^, Ery^r^	17
pGCC4-*mtrD*_6His_	*mtrD* cloned into PmeI and PacI sites of pGCC4 under the control of P*_lac_* promoter with a 6His tag at the C terminus	This study
pGCC4-CL*mtrD*_His6_	Cysteineless derivative of *mtrD*_His6_ in pGCC4	This study
pGCC4-CL*mtrD*_His6_(F136A)	Site-directed F136A MtrD mutant based on pGCC4-CL*mtrD*_His6_	This study
pGCC4-CL*mtrD*_His6_(F176A)	Site-directed F176A MtrD mutant based on pGCC4-CL*mtrD*_His6_	This study
pGCC4-CL*mtrD*_His6_(I605A)	Site-directed I605A MtrD mutant based on pGCC4-CL*mtrD*_His6_	This study
pGCC4-CL*mtrD*_His6_(F610A)	Site-directed F610A MtrD mutant based on pGCC4-CL*mtrD*_His6_	This study
pGCC4-CL*mtrD*_His6_(S611A)	Site-directed S611A MtrD mutant based on pGCC4-CL*mtrD*_His6_	This study
pGCC4-CL*mtrD*_His6_(F612C)	Site-directed F612C MtrD mutant based on pGCC4-CL*mtrD*_His6_	This study
pGCC4-CL*mtrD*_His6_(F623C)	Site-directed F6123C MtrD mutant based on pGCC4-CL*mtrD*_His6_	This study

a Abbreviations: Ery^r^, erythromycin resistance; Kan^r^, kanamycin resistance.

E. coli DH5α (42) was used in cloning experiments and for propagation of all pGCC4 vector-based constructs. E. coli cells were grown on LB agar or in LB broth (Oxoid; Thermo Fisher Scientific Australia Pty. Ltd.) with kanamycin (40 μg/ml) where required.

### Molecular biology methods.

Chromosomal DNA was isolated from N. gonorrhoeae cells by the use of a Wizard genomic DNA purification kit (Promega Co., USA) and plasmid DNA from E. coli using an Isolate II plasmid minikit (Bioline, Boston, USA) per the instructions of the manufacturers. Primers used in this study are listed in Table S1 in the supplemental material and were synthesized by GeneWorks (GeneWorks Pty. Ltd., Australia) or Integrated DNA Technologies. Bioline Velocity DNA polymerase was used in all cloning and mutagenesis procedures and Bioline Mango*Taq* DNA polymerase for screening PCRs (Bioline, Boston, MA, USA). All restriction digestion enzymes and T4 DNA ligase were from New England Biolabs (New England Biolabs Inc., Ipswich, MA, USA). Transformations into N. gonorrhoeae or E. coli cells were carried as described previously (42, 43). All sequencing was performed by Australian Genome Research Facility (AGRF; Australia).

10.1128/mBio.02277-19.7 TABLE S1 Oligonucleotides used in this study. Download Table S1, DOCX file, 0.02 MB. Copyright © 2019 Chitsaz et al. 2019 Chitsaz et al. This content is distributed under the terms of the Creative Commons Attribution 4.0 International license.

### Construction of unmarked *mtrD* and *norM* single and *mtrD*/*norM* double deletion strains.

Two unmarked *mtrD* deletion strains (the FA19 Δ*mtrD* and KH15 Δ*mtrD* strains) were created by overlap extension PCR (12). Briefly, a 3,150-bp fusion DNA fragment that encompassed the *mtrD* gene was created by PCR using primers MtrD-Del-F2, MtrD-Ovl-R1, MtrD-Ovl-F2, and MtrD-Del-R2 (Table S1) and FA19 chromosomal DNA as the template. This two-step PCR removed *mtrD*, leaving only 177 bp before its stop codon. The PCR-amplified DNA was transformed into the FA19 and KH15 strains, and the resultant colonies were selected by analysis of susceptibility to erythromycin performed by replica plating on GC plates with or without erythromycin (0.06 μg/ml, 4-fold below MIC for FA19). Erythromycin was used as it is a known substrate of the MtrD protein, and sensitivity to erythromycin at a concentration below the MIC for FA19 could be used as evidence that the *mtrD* gene had been removed. Sequencing confirmed the removal of *mtrD* and the integrity of the flanking regions. The same method was used for creating the N. gonorrhoeae FA19 Δ*norM*, FA19 Δ*mtrD* Δ*norM*, and KH15 Δ*mtrD* Δ*norM* strains by deleting the *norM* gene from the respective progenitors. The NorM substrate ethidium (0.25 μg/ml for *norM*-only deletion and 0.06 μg/ml for *norM* and *mtrD* double deletion mutants) was used for selecting the *norM* knockout derivatives by replica plating.

### Cloning of *mtrD* into pGCC4 and recombination into the neisserial chromosome.

To recombine *mtrD* into the chromosome of the N. gonorrhoeae Δ*mtrD* knockout strains, the *mtrD* determinant was cloned into the pGCC4 shuttle vector behind the lac promoter. A PacI-*mtrD*-PmeI fragment was obtained by PCR amplification using FA19 chromosomal template DNA and PacI-*mtrD*-for and PmeI-*mtrD*-rev primers (Table S1). A sequence was also incorporated in the reverse primer, allowing addition of six histidine residues (His6) to act as an affinity tag at the C terminus of the protein. The resultant PCR product and vector were digested and cloned with PacI and PmeI, producing the *mtrD* clone called pGCC4-*mtrD*_(His6)_.

The *mtrD* derivatives were recombined into the KH15 Δ*mtrD* Δ*norM* chromosome by transformation as previously described (43). The pGCC4 empty vector was also integrated into the KH15 Δ*mtrD* Δ*norM* chromosome, producing a NICS-only recombinant strain that could be utilized as a negative control by employment of the same methodology. KH15 Δ*mtrD* Δ*norM* recombinants were selected on GC plates containing erythromycin (0.5 μg/ml). A single purified transformant colony was subjected to PCR using SCRNG1 and MTRDSF5 primers (Table S1) for verification of *mtrD*_His6_ integration between *lctP* and *aspC*.

### Site-directed mutagenesis.

The QuikChange site-directed mutagenesis method was used to individually replace the selected MtrD residues using the primers listed in Table S1. To aid in screening, a silent restriction site was incorporated into each primer pair where possible. Initially, a cysteineless derivative of MtrD was created by PCR amplification using C491A-F and C491A-R primers and pGCC4-*mtrD*_His6_ as the template. The PCR cycling conditions were as follows: denaturation at 95°C for 5 min, followed by 30 cycles of 95°C for 20 s, 55°C for 1 min, 72°C for 8 min 30 s and then 72°C for 10 min. Subsequent mutants (F136A, F176A, I605A, F610A, S611A, F612C, and F623C) were created using the pGCC4-CL*mtrD*_His6_ construct as the template. The integrity of all constructs was verified by sequencing the whole *mtrD* gene followed by recombination of the gene into the N. gonorrhoeae KH15 Δ*mtrD* Δ*norM* chromosome.

### Western blotting.

Expression of MtrD proteins in KH15 Δ*mtrD* Δ*norM* membranes was analyzed by Western blotting using anti-6×His epitope tag (rabbit) antibody and peroxidase-conjugated anti-rabbit IgG (goat) antibody (Rockland) as primary and secondary antibodies, respectively. N. gonorrhoeae strains were grown at 37°C to an optical density at 600 nm (OD_600_) of 0.6 in GC broth containing 0.5 mM IPTG. All membrane isolation and protein experiments were conducted on ice or at 4°C as previously described (44, 45). The total protein content in each sample was quantified using a Bio-Rad DC protein assay kit, and 10-μg samples were resolved on a 10% SDS-PAGE gel and transferred (44, 45). Membranes were scanned with a Bio-Rad ChemiDoc MP imaging system and analyzed using Image Lab software 6.0.1 (Bio-Rad). Mutant protein expression levels were compared to the WT protein expression level (see Fig. S1 in the supplemental material).

### MIC analyses.

MIC analyses of the N. gonorrhoeae strains used a previously reported agar dilution method (46) with modifications. MIC analyses were conducted using solid GC media supplemented with 0.5 mM IPTG and antimicrobial compounds. The MIC was determined as the lowest concentration of the antimicrobial compound required to fully inhibit bacterial growth. All MIC values shown are representative of results from three replicates.

### Molecular dynamics simulations.

The MtrD crystal structure (PDB identifier [ID]: 4MT1) was used for all simulations (7). The missing residues between residues 494 and 507, 671, and 672 were rebuilt; the N and C termini were capped with acetyl and amine groups, respectively; and the crystallographic symmetry operators were applied to rebuild the trimeric biological assembly (47). The MtrD trimer was embedded in an equilibrated *Neisserial* lipid bilayer containing 80% 1,2-dipalmitoyl-*sn*-glycero-3-phosphoethanolamine (DMPE) and 20% 1,2-dimyristoyl-sn-glycero-3-phospho-(1′-rac)-glycerol (DMPG) as described previously (48), using the Orientation of Membrane Proteins database to guide the orientation in the membrane (49). Thirty molecules of progesterone were inserted randomly in the solvent layer surrounding MtrD. The system was solvated with explicit water and 150 mM NaCl. Counter-ions were added to ensure the overall charge neutrality of the system.

All simulations were performed using GROMACS 2016.1 (50, 51) in conjunction with the GROMOS 54a7 forcefield (52) and a 2-fs time step. The simple point charge (SPC) water model (53) was used to describe the solvent water. The parameters used for the progesterone analyses, DMPE and DMPG, are available from the automated force field topology builder (ATB) (54). All simulations were performed under periodic boundary conditions in a hexagonal prism box. The dimensions of the box were chosen such that minimum distance of the protein to its periodic image was 3.5 nm.

The system was equilibrated over 10 ns, using a series of 2-ns simulations in the NPT (fixed number of atoms N, fixed pressure P, fixed temperature T) ensemble with progressively decreasing position restraint force constants of 500 kJ mol^−1 ^nm^−2^, 100 kJ mol^−1 ^nm^−2^, 50 kJ mol^−1 ^nm^−2^, 20 kJ mol^−1 ^nm^−2^, and 0 kJ mol^−1 ^nm^2^ on the Cα atoms. The temperature of the simulations was maintained at 300 K using a Bussi velocity rescaling thermostat, with a coupling constant of 0.1 ps (55). The pressure coupling was semi-isotropic. The pressure was maintained at 1 bar using a Parrinello-Rahman barostat pressure coupling with a constant of 5 ps. The Particle Mesh Ewald (PME) method (56) was used to calculate electrostatic interactions with a cutoff at 1.4 nm. The LINCS algorithm (57) was used for bond constraints, and the SETTLE algorithm (58) was utilized to constrain waters. Three replicate simulations were performed, each 200 ns in length. To examine how changes in timescale influence the interaction with progesterone, the third simulation was extended to 500 ns in total, giving a total combined simulation time of 900 ns for the three replicates.

### Docking studies.

To identify the molecular interactions underlying the modified resistance profiles of our MtrD mutants, molecular docking of seven diverse MtrD substrates (progesterone, nonoxynol-9, azithromycin, rifampin, ethidium, crystal violet, and cholic acid) and the PAβN inhibitor was performed with AutoDock Vina V.1.1.2. United-atom representations of these substrates (with protonation states appropriate for pH 7.4) were docked to the repaired crystal structure of the MtrD trimer used to initialize the molecular dynamics simulations reported in this study. AutoDock Tools V.1.5.6 was used to determine two sets of grid parameters centered on the access and deep binding pocket cavities, respectively (Table S2). For each substrate, docked poses within the proposed access pocket or deep binding pocket were selected for analysis if the energy of a given pose was within 1 kcal/mol (inclusive) of the lowest-energy pose for a given substrate, with weighted energies obtained according to the default AutoDock Vina scoring function. Substrate amide bonds were allowed to rotate during docking. Residues comprising the putative access and deep binding pockets (Table 4) were made flexible during the docking procedure centered on the corresponding pocket. To identify the residues with the most significant binding interactions, approximate per-residue contributions to the binding affinity and energy breakdowns for each substrate were obtained by systematically removing each residue found within 8 Å of the lowest-scoring docked pose followed by (static) rescoring.

10.1128/mBio.02277-19.8 TABLE S2 Docking parameters used. Download Table S2, DOCX file, 0.02 MB. Copyright © 2019 Chitsaz et al. 2019 Chitsaz et al. This content is distributed under the terms of the Creative Commons Attribution 4.0 International license.

10.1128/mBio.02277-19.4 FIG S4 (A) The lowest-energy docked poses for nonoxynol-9 in the access pocket. F612 consistently interacts with nonoxynol-9 in the lowest-energy docked poses. (B) The lowest-energy docked poses for cholic acid. Here, F610, F612, F136, and R174 (magenta sticks) interact with the lowest-energy conformations of cholic acid docked to either the access pocket or the deep binding pocket of MtrD. Download FIG S4, DOCX file, 2.4 MB. Copyright © 2019 Chitsaz et al. 2019 Chitsaz et al. This content is distributed under the terms of the Creative Commons Attribution 4.0 International license.

10.1128/mBio.02277-19.5 FIG S5 (A) The lowest-energy docked poses for rifampin. Docking results suggest that F612 (omitted for clarity) plays a key role in rifampicin binding. (B) The lowest-energy docked poses for azithromycin. Residues F136, R174, F610, and F612 (magenta sticks) interact with the lowest-energy docked poses of azithromycin. Download FIG S5, DOCX file, 1.3 MB. Copyright © 2019 Chitsaz et al. 2019 Chitsaz et al. This content is distributed under the terms of the Creative Commons Attribution 4.0 International license.

10.1128/mBio.02277-19.6 FIG S6 The lowest-energy docked poses for PAβN. Here, F610, F612, and F136 (magenta sticks) interact with the lowest-energy docked poses of PAβN. Download FIG S6, DOCX file, 0.4 MB. Copyright © 2019 Chitsaz et al. 2019 Chitsaz et al. This content is distributed under the terms of the Creative Commons Attribution 4.0 International license.
