# Supplementary material for: Multidrug Resistance in Neisseria gonorrhoeae: Identification of Functionally Important Residues in the MtrD Efflux Protein
Source: mBio. 2019 Nov 19;10(6):e02277-19. doi: 10.1128/mBio.02277-19 (PMC6867893; doi:10.1128/mBio.02277-19)
Supplement: FIG S2 [file mBio.02277-19-sf002.docx]

**
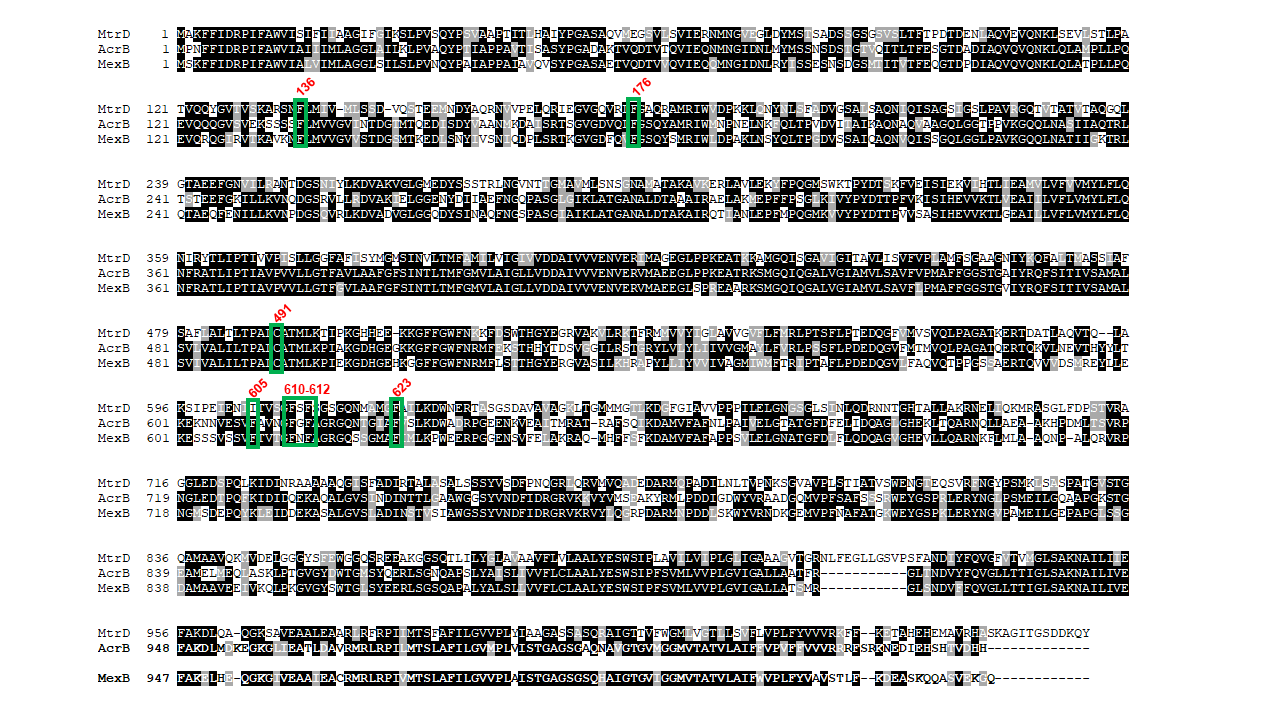
**

**FIG S2.** Protein sequence alignment of MtrD (Accession no AAC45560.1), AcrB (Accession no P31224) and MexB (Accession no P52002). Protein sequence alignment was done using the T-Coffee program and visualized by BoxShade with residue boxes colored based on % identity. MtrD residues targeted by site-directed mutagenesis are shown within green rectangles with residue number at the top in red.
