# Supplementary material for: Multidrug Resistance in Neisseria gonorrhoeae: Identification of Functionally Important Residues in the MtrD Efflux Protein
Source: mBio. 2019 Nov 19;10(6):e02277-19. doi: 10.1128/mBio.02277-19 (PMC6867893; doi:10.1128/mBio.02277-19)
Supplement: FIG S1 [file mBio.02277-19-sf001.docx]

**
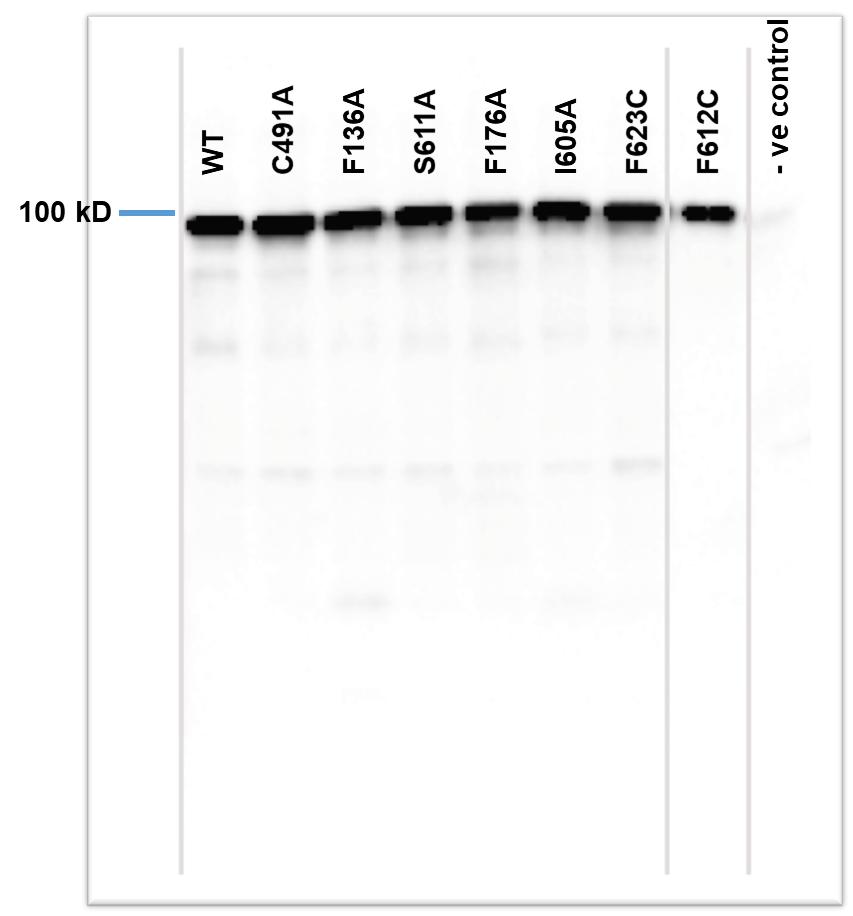
**

**FIG S1.** An example Western blot showing expression levels of the recombinant wild-type (WT) MtrD and seven MtrD mutants. Membrane protein samples are of isolated membranes from the KH15*∆mtrD*∆*norM* strain expressing these MtrD mutant derivatives. The negative (–ve) control is KH15*∆mtrD*∆*norM* complemented with a Not I digested NICS portion of the pGCC4 vector, lacking the *mtrD* gene. After transfer to a polyvinylidene difluoride membrane, MtrD proteins were immunologically detected using a rabbit anti-6 his tag antibody that binds to the C-terminal histag on MtrD.
