# Supplementary material for: Multidrug Resistance in Neisseria gonorrhoeae: Identification of Functionally Important Residues in the MtrD Efflux Protein
Source: mBio. 2019 Nov 19;10(6):e02277-19. doi: 10.1128/mBio.02277-19 (PMC6867893; doi:10.1128/mBio.02277-19)
Supplement: FIG S3 [file mBio.02277-19-sf003.docx]

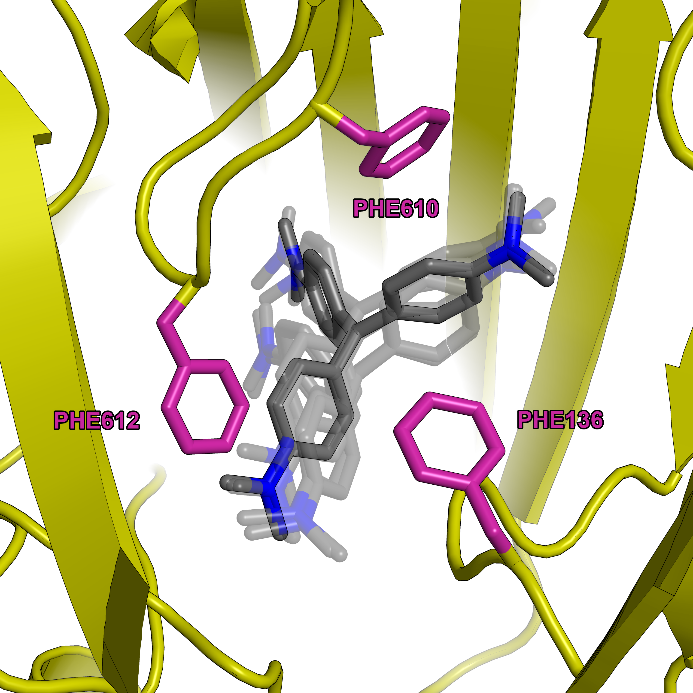

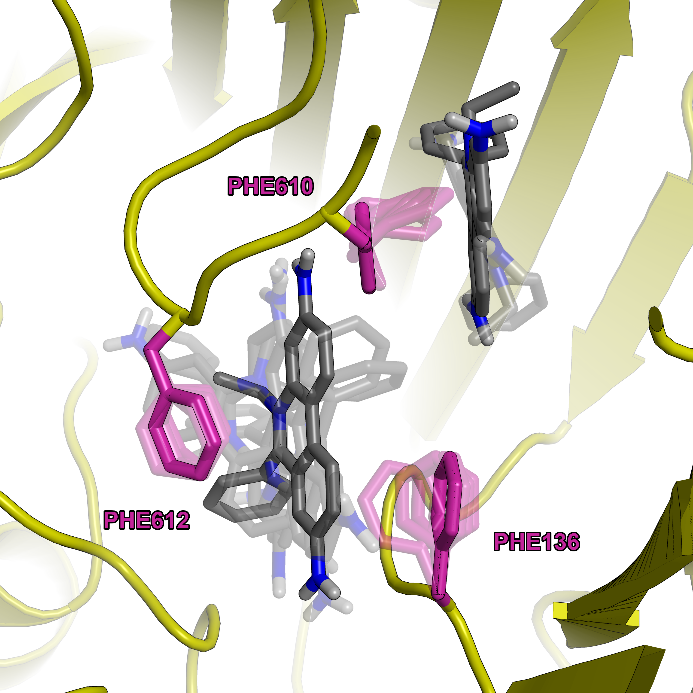


**A**

**B**

**FIG S3.** A) The lowest-energy docked poses for crystal violet. Three residues, F612, F136, and F610 (magenta sticks), form key interactions with crystal violet in all of the lowest energy docked conformations. B) The lowest-energy docked poses for ethidium. Residues F610, F612 and F136 (magenta sticks) interact with the lowest energy conformations of ethidium docked to either the access or deep binding pocket of MtrD.
