# Supplementary material for: Multidrug Resistance in Neisseria gonorrhoeae: Identification of Functionally Important Residues in the MtrD Efflux Protein
Source: mBio. 2019 Nov 19;10(6):e02277-19. doi: 10.1128/mBio.02277-19 (PMC6867893; doi:10.1128/mBio.02277-19)
Supplement: TABLE S1 [file mBio.02277-19-st001.docx]

**Table S1.** Oligonucleotides used in this study

| **Name and purpose** | **Sequence 5' to 3'** | **Source** |
| --- | --- | --- |
| Cloning *mtrD* into pGCC4 | |  |
| PacI-mtrD for | GAGATTAATTAA AGGAAGGCATCGATGGCTAAATT | This study |
| PmeI-mtrD rev | TCTCGTTTAAACTTAATGGTGATGATGGTGATGATATTGTTTATCGTCCG | This study |
| PCR screening for integration of *mtrD* between *acpC* and *lctP* |  |  |
| SCRNG1 | CGCCTTATGATGCAAATG | This study |
| MTRDSF5 | CCGTATTCTGGGGGATGTTG | This study |
| Sequencing of *mtrD* in pGCC4 |  |  |
| PGCC4_F2 | CCCAGGCTTTACACTTTATGCT | This study |
| PGCC4-R1.1 | GGGGATCCGCTAGCACTAGG | This study |
| MtrD-SF4.1 | GCAGTTGGGTACGGCAGAAG | This study |
| MtrD-SR4.1 | GCTCTGTTCCGTACCGTTTTC | This study |
| Deleting *mtrD* from *N. gonorrhoeae* chromosome |  |  |
| MtrD-Del-F1 | CGCGTCAAACAAGTCTTC | This study |
| MtrD-Del-F2 | GGCAAAACCCTTTACATC | This study |
| MtrD-Ovl-R1 | GGTATTTTGCCGATGCCTTCCTTATTTCGCTTC | This study |
| MtrD-Ovl-F2 | GGAAGGCATCGATCGGCACGACCGTATTCTG | This study |
| MtrD-Del-R2 | GGCAAAACCCTTTACATC | This study |
| Deleting *norM* from *N. gonorrhoeae* chromosome |  |  |
| NorM-AF1 | GCAGCGAGAATGCTTTTG | This study |
| NorM-AR | GCAATCAATGCCGTCAAGTGTTCAGTCAATAAAAATG | This study |
| NorM-BF | GACTGAACACTTGACGGCATTGATTGC | This study |
| NorM-BR1 | CCAATGCCTGTTTATCGTG | This study |
| Mutagenesis |  |  |
| C491A-F | CTGACCCTTACCCCTGCGCTAGCAGCCACAATGTTGAAG | This study |
| C491A-R | CTTCAACATTGTGGCTGCTAGCGCAGGGGTAAGGGTCAG | This study |
| F136A-F | CAAGGCGCGTTCCAATGCATTGATGATTGTGATG | This study |
| F136A-R | CATCACAATCATCAATGCATTGGAACGCGCCTTG | This study |
| F176A-F | CAGGTCCGCCTGGCTGGGGCGCAACGCGCGATG | This study |
| F176A-R | CATCGCGCGTTGCGCCCCAGCCAGGCGGACCTG | This study |
| I605A-F | CGAAATTGAAAACATCGCGACCGTTTCCGGCTTC | This study |
| I605A-R | GAAGCCGGAAACGGTCGCGATGTTTTCAATTTCG | This study |
| F610A-F | CGTTTCCGGCGCTAGCTTTTCGGGCAGCGGTCAG | This study |
| F610A-R | CTGACCGCTGCCCGAAAAGCTAGCGCCGGAAACG | This study |
| S611A-F | CGTTTCCGGCTTCGCGTTTTCGGGCAGCGGTCAG | This study |
| S611A-R | CTGACCGCTGCCCGAAAACGCGAAGCCGGAAACG | This study |
| F612C-F | CGTTTCCGGCTTCAGCTGCTCAGGCAGCGGTCAGAATATG | This study |
| F612C-R | CATATTCTGACCGCTGCCTGAGCAGCTGAAGCCGGAAACG | This study |
| F623C-F | CAGAATATGGCGATGGGTTGCGCAATATTGAAAGATTGGAAC | This study |
| F623C-R | GTTCCAATCTTTCAATATTGCGCAACCCATCGCCATATTCTG | This study |
