# Supplementary material for: Multidrug Resistance in Neisseria gonorrhoeae: Identification of Functionally Important Residues in the MtrD Efflux Protein
Source: mBio. 2019 Nov 19;10(6):e02277-19. doi: 10.1128/mBio.02277-19 (PMC6867893; doi:10.1128/mBio.02277-19)
Supplement: TABLE S2 [file mBio.02277-19-st002.docx]

**Table S2.** Docking parameters used

| **Parameter** | **Access Pocket Grid** | **Deep Binding Pocket Grid** |
| --- | --- | --- |
| **X centre** | 26.061 | 25.725 |
| **Y centre** | -17.867 | -8.799 |
| **Z centre** | 40.542 | 47.640 |
| **X points** | 46 | 44 |
| **Y points** | 46 | 44 |
| **Z points** | 46 | 44 |
| **Spacing (Angstroms)** | 0.375 | 0.375 |
| **Exhaustiveness** | 12 | 12 |
