# Supplementary material for: Multidrug Resistance in Neisseria gonorrhoeae: Identification of Functionally Important Residues in the MtrD Efflux Protein
Source: mBio. 2019 Nov 19;10(6):e02277-19. doi: 10.1128/mBio.02277-19 (PMC6867893; doi:10.1128/mBio.02277-19)
Supplement: FIG S4 [file mBio.02277-19-sf004.docx]

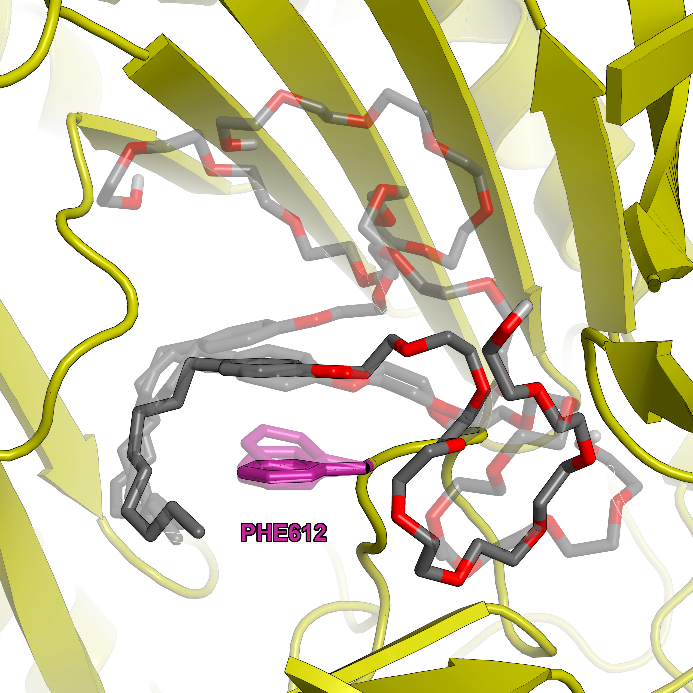

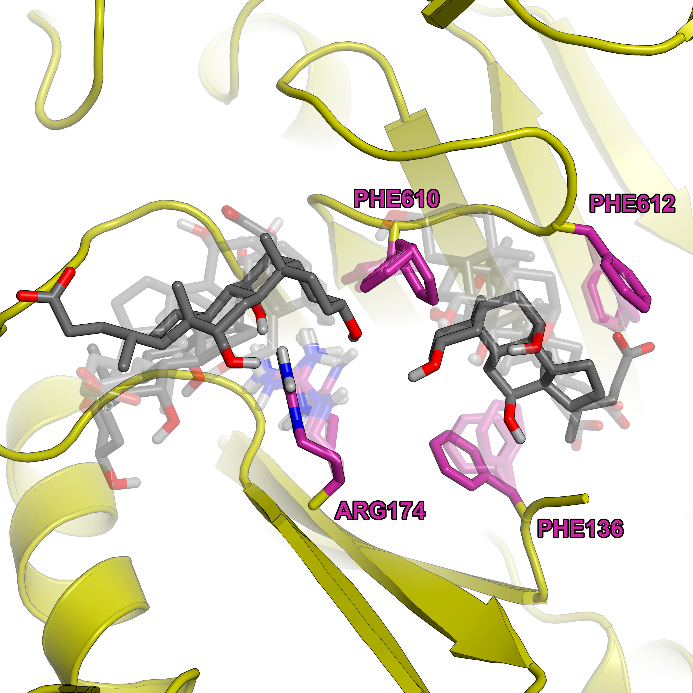


**A**

**B**

**FIG S4.** A) The lowest-energy docked poses for nonoxynol-9 in the access pocket. F612 consistently interacts with nonoxynol-9 in the lowest energy docked poses. B) The lowest-energy docked poses for cholic acid. Here F610, F612, F136, and R174 (magenta sticks) interact with the lowest energy conformations of cholic acid docked to either the access or deep binding pocket of MtrD.
