# Supplementary material for: Multidrug Resistance in Neisseria gonorrhoeae: Identification of Functionally Important Residues in the MtrD Efflux Protein
Source: mBio. 2019 Nov 19;10(6):e02277-19. doi: 10.1128/mBio.02277-19 (PMC6867893; doi:10.1128/mBio.02277-19)
Supplement: FIG S5 [file mBio.02277-19-sf005.docx]

**A B**


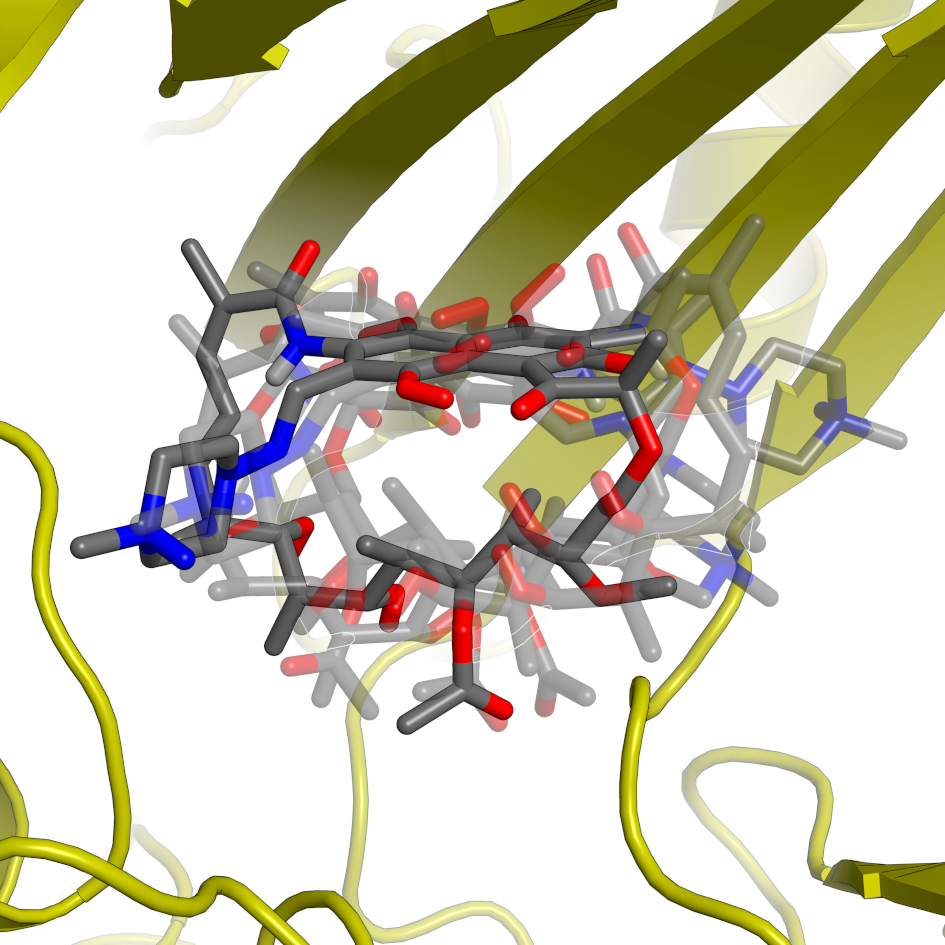
i
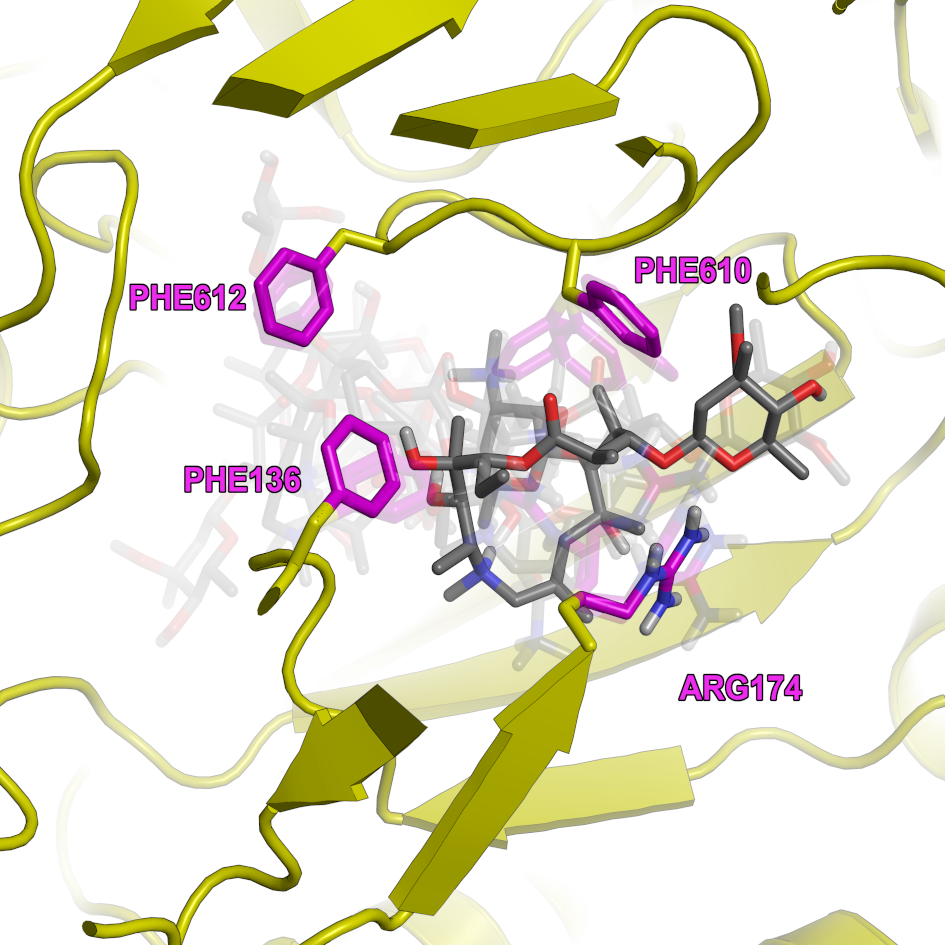
 **FIG S5.** A) The lowest-energy docked poses for rifampin. Docking results suggest that F612 (not shown for clarity) plays a key role in rifampicin binding. B) The lowest-energy docked poses for azithromycin. Residues F136, R174, F610 and F612 (magenta sticks) interact with the lowest energy docked poses of azithromycin.
