# Supplementary material for: Multidrug Resistance in Neisseria gonorrhoeae: Identification of Functionally Important Residues in the MtrD Efflux Protein
Source: mBio. 2019 Nov 19;10(6):e02277-19. doi: 10.1128/mBio.02277-19 (PMC6867893; doi:10.1128/mBio.02277-19)
Supplement: FIG S6 [file mBio.02277-19-sf006.docx]

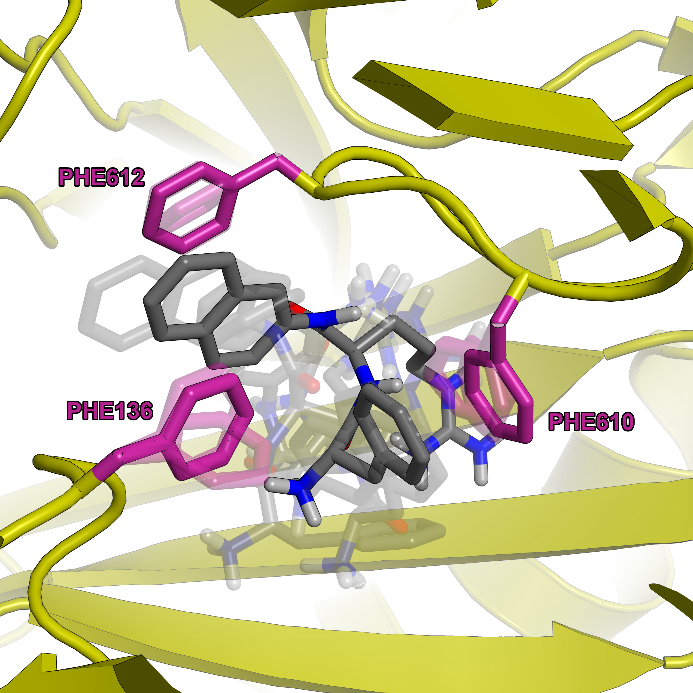


**FIG S6.** The lowest-energy docked poses for PAβN. Here F610, F612 and F136 (magenta sticks) interact with the lowest energy docked poses of PAβN.
